# Supplementary figures and images for: Sucrose-delaying flower color fading associated with delaying anthocyanin accumulation decrease in cut chrysanthemum
Source: PeerJ. 2023 Dec 11;11:e16520. doi: 10.7717/peerj.16520 (PMC10720401; doi:10.7717/peerj.16520)

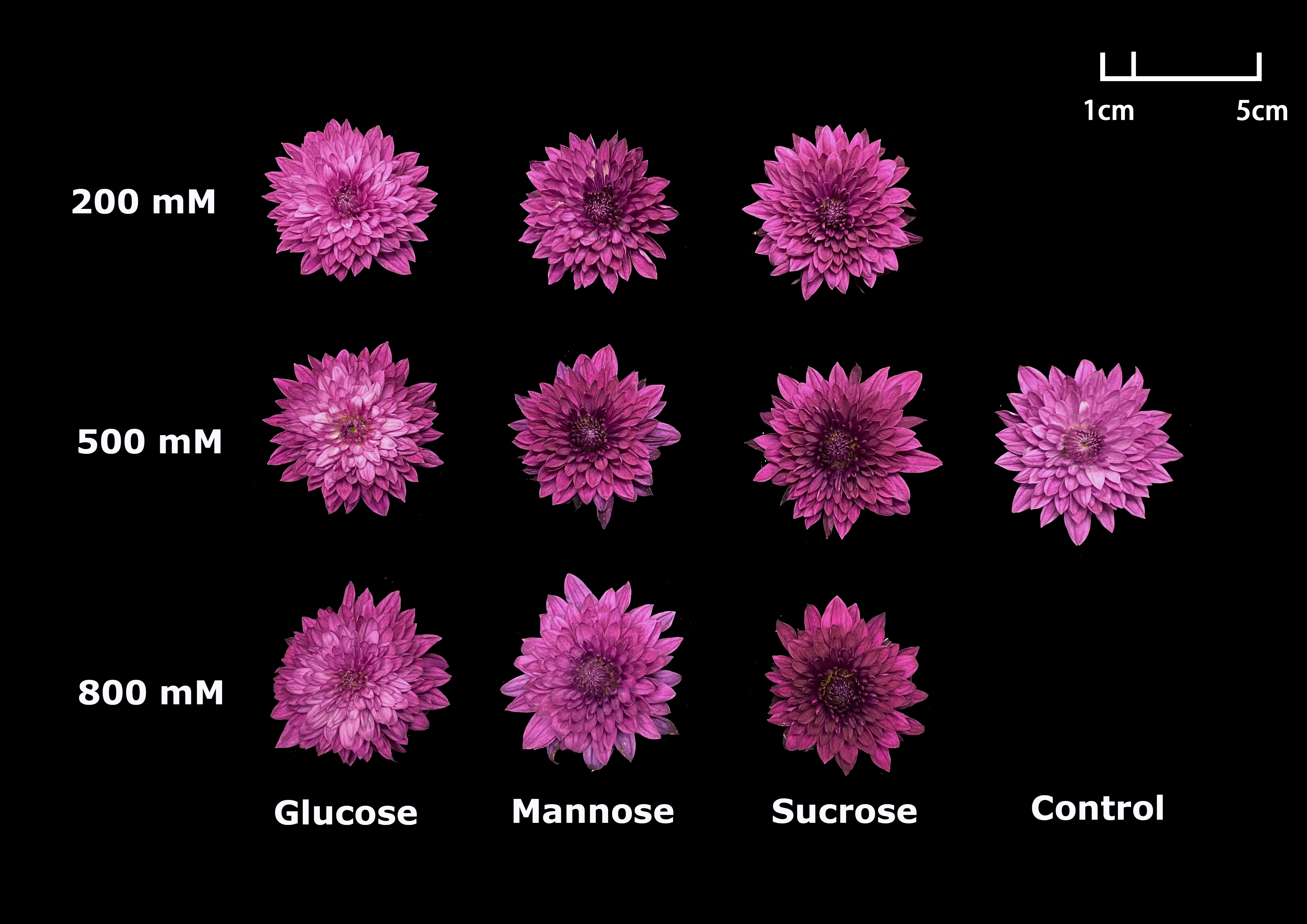

Supplement: Supplemental Information 1 [file peerj-11-16520-s001.jpg]

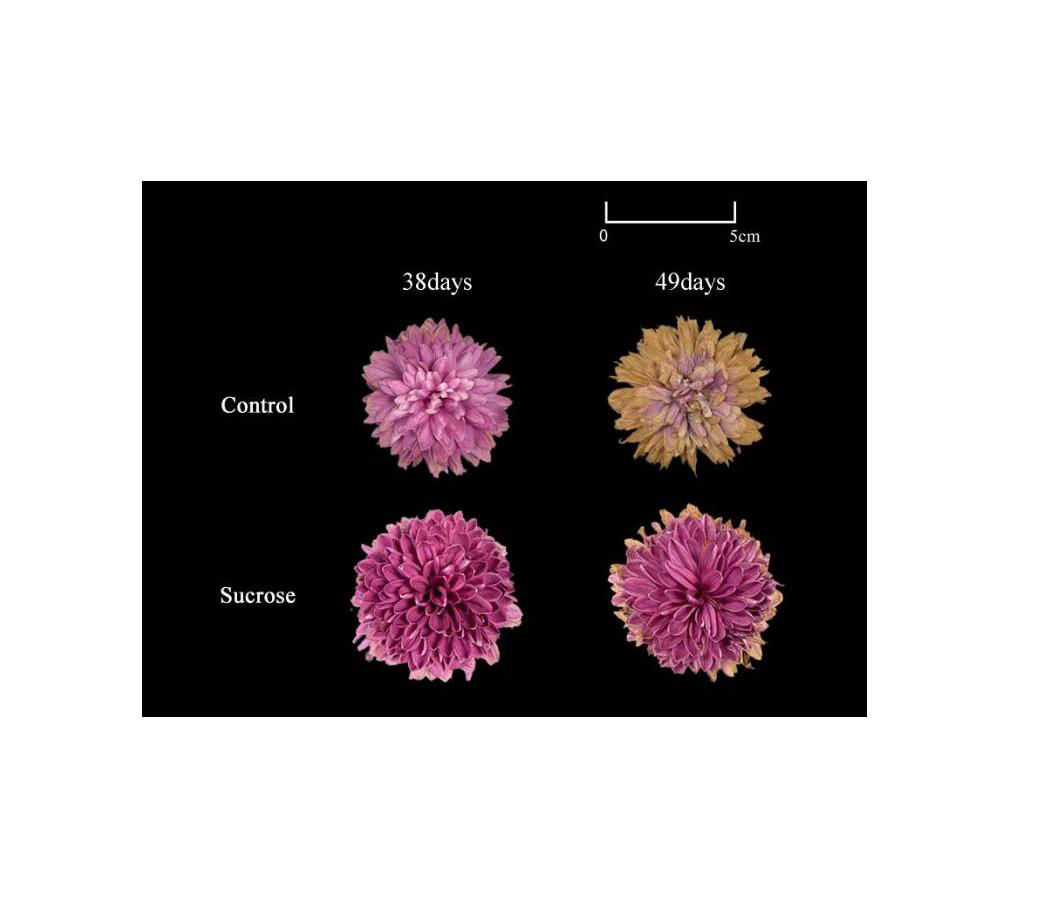

Supplement: Supplemental Information 2 [file peerj-11-16520-s002.png]
